# Enhanced Human Activity Recognition Using Wearable Sensors via a Hybrid Feature Selection Method

**DOI:** 10.3390/s21196434

**Published:** 2021-09-26

**Authors:** Changjun Fan, Fei Gao

**Affiliations:** College of Computer Science and Technology, Zhejiang University of Technology, Hangzhou 310023, China; cjfan@zju.edu.cn

**Keywords:** bee swarm optimization, deep Q-network, feature selection, human activity recognition, hybrid metaheuristic, wearable sensors, multi-agent reinforcement learning

## Abstract

The study of human activity recognition (HAR) plays an important role in many areas such as healthcare, entertainment, sports, and smart homes. With the development of wearable electronics and wireless communication technologies, activity recognition using inertial sensors from ubiquitous smart mobile devices has drawn wide attention and become a research hotspot. Before recognition, the sensor signals are typically preprocessed and segmented, and then representative features are extracted and selected based on them. Considering the issues of limited resources of wearable devices and the curse of dimensionality, it is vital to generate the best feature combination which maximizes the performance and efficiency of the following mapping from feature subsets to activities. In this paper, we propose to integrate bee swarm optimization (BSO) with a deep Q-network to perform feature selection and present a hybrid feature selection methodology, BAROQUE, on basis of these two schemes. Following the wrapper approach, BAROQUE leverages the appealing properties from BSO and the multi-agent deep Q-network (DQN) to determine feature subsets and adopts a classifier to evaluate these solutions. In BAROQUE, the BSO is employed to strike a balance between exploitation and exploration for the search of feature space, while the DQN takes advantage of the merits of reinforcement learning to make the local search process more adaptive and more efficient. Extensive experiments were conducted on some benchmark datasets collected by smartphones or smartwatches, and the metrics were compared with those of BSO, DQN, and some other previously published methods. The results show that BAROQUE achieves an accuracy of 98.41% for the UCI-HAR dataset and takes less time to converge to a good solution than other methods, such as CFS, SFFS, and Relief-F, yielding quite promising results in terms of accuracy and efficiency.

## 1. Introduction

In daily life, human beings keep performing all kinds of activities, such as walking, typing, eating, smoking and so on, and it is of great significance to explore these behaviors. Human activity recognition (HAR) is a hot but challenging research topic. It aims at using some technical means to determine the activity patterns or types of a person [1]. This is crucial for a wide range of applications, such as long-term healthcare monitoring [2,3], active and assisted living systems [4], monitoring and surveillance systems [5], and smart homes [6].

The earliest research on HAR can be traced back to the 1990s when Foerster et al. [7] collected data from an accelerometer in a laboratory environment to assess tremor activity as well as to detect posture and motion. Since then, human activities have often been investigated by inertial sensor-based systems using accelerometers, gyroscopes, and other sensors. With the rapid development of MEMS technology, these sensors are embedded into wearable devices thanks to their decrease in size and the increase in precision [8]. Smartphones and smartwatches (or wristbands) are two typical examples of popular wearable smart devices [9]. They both usually come with a wealth of sensors to facilitate a better user experience, including accelerometers, gyroscopes, magnetometers, and some others, like GPS, light intensity. Nowadays, these off-the-shelf commercial devices are becoming easily available, increasingly popular and an indispensable part of our daily lives. Such wearable devices are lightweight, portable, and unobtrusive with low power consumption, which makes them suitable and convenient to monitor daily activities for people. Based on such wearable devices and wireless communication technology, a wireless body area network (WBAN) can be deployed to track users’ everyday activities [10], which brings unlimited possibilities for online or offline HAR applications.

Human activity recognition is essentially a supervised classification problem. The data collected from the wearable sensors is processed and then mapped to a predefined set of activities, like walking, writing, or smoking. Although a motion sensor can obtain the local inertial information of one’s body, its readings are abstract and uninterpretable, and there exists a gap between these raw data and the corresponding activity being performed. Therefore, it is intractable to be fully aware of what’s going on in terms of the motion context of the whole body. In order to fulfill the mapping, the usual practice is to extract as many features from the sensor data as possible to represent the corresponding activity and then select the most relevant ones to ensure a good recognition performance.

When carrying out classification, machine learning-based methods require a very large amount of data. And the data required for an accurate recognition increases dramatically as the number of feature dimensions increases, which tends to cause the “curse of dimensionality” problem and ends up with degraded classification performance. Unlike cameras or binary sensors, the readings of wearable sensors are not visually intuitive to people, and manually labeling a great number of different types of daily activities is labor intensive or even infeasible, which makes it very difficult to obtain sufficient labeled data. Furthermore, not all the features have the same representation ability. Some of them make a great contribution to enhance the following classification performance, while some others may be irrelevant, or even redundant, to human activity recognition tasks, which deteriorates the classification performance badly. Therefore, it is critical to keep the more representative features in and remove the others.

Generally, WBAN-based HAR applications have a multi-layer framework and mainly involve communication and computation. For example, in a WBAN consisting of a smartphone and smartwatch, the smartphone plays the role of a computing center and route hub due to its remarkable computing and communication capacities, while both the smartwatch and smartphone act as a sensor node. In the online scenario, model inference may be performed directly on the phone, and features may be extracted on the watch locally and then transmitted to the phone for fusion. Such mobile devices have limited resources, like battery capacity, memory, and computing resources. More features to extract mean more computing and communication operations carried out on the mobile devices, and more features mean more computational cost for classification. It speeds up power consumption and reduces standby time for these devices.

In this study, to address the aforementioned issues, we propose a hybrid feature selection method, BAROQUE, to enhance human activity recognition based on wearable sensors in a smartphone or/and smartwatch. To search through the feature space, the biggest challenge is that the space increases exponentially with respect to the number of features. Considering this, BAROQUE integrates bee swarm optimization (BSO) metaheuristic with a multi-agent deep Q-network to ensure a good performance. Among these, the BSO implements the intensification and diversification mechanisms, which guarantee a good balance between exploitation and exploration of the search space; and the DQN takes advantage of the merits of reinforcement learning (RL) to make the local search process for each agent more adaptive and more efficient.

This work is organized as follows: Section 2 introduces previous related studies and their outcome. Section 3 describes in detail human activity recognition system architecture with focus on the proposed hybrid feature selection method. Section 4 presents the experimental setup for evaluating the proposed approach, and the corresponding results are provided and analyzed in this section too. Section 5 concludes this research with future scopes.

## 2. Related Works

Some recent research works [11,12,13,14] presented a detailed review of human activity recognition solutions based on wearable sensors from different angles, involving the adopted sensors, recognition approaches, and application scenarios. From these, we can see that inertial sensors, especially accelerometers, are the most commonly used wearable sensors for action/activity recognition due to their ability to measure attributes related to the user’s movement. Bao et al. [15] used five two-axis accelerometers worn on the user’s right hip, dominant wrist, non-dominant upper arm, dominant ankle, and non-dominant thigh to recognize 20 different activities using decision tables, instance-based learning, C4.5, and naïve Bayes classifiers. They claimed that a recognition accuracy of over 80% on a variety of 20 everyday activities was achieved and multiple accelerometers aided in recognition. This study was one of the most classic works in the early days of HAR research based on wearable sensors, and the framework it proposed became an important reference for follow-up research works.

Since then, some similar HAR systems, requiring users to wear four or more accelerometers [16,17], or carry heavy recording devices [18], were presented. However, wearing too many devices is obtrusive and intrusive and can cause additional burdens to users. As smartphones are increasingly popular, they become an integral part of people’s daily life. A growing number of studies began to adopt smartphones for context-aware activity recognition in pervasive and ubiquitous environments, benefitting from their embedded various sensors and lightweight, portable characteristics. The research works [19,20,21,22,23,24,25,26,27] presented different approaches to recognize various human activities using smartphone sensors. Additionally, Paul et al. [28] took the sensor readings from mobile sensors as inputs and predicted a human motion activity using online classification algorithms; Cao et al. [29] proposed an efficient group-based context-aware classification method for human activity recognition on smartphones. These two studies carried out online activity recognition on smartphones using the embedded sensors, thanks to their ever-growing computing, networking, and sensing abilities. Likewise, as another kind of commonly used wearable device, wrist-worn devices represented, for example, by smartwatches or wristbands, are employed to provide HAR solutions using their embedded accelerometers, gyroscopes, magnetometers, or even heart rate monitors. For example, Mekruksavanich et al. [30] suggested the use of data from the accelerometer and gyroscope in a smartwatch to detect sitting for curbing the sedentary habit. Kwon et al. [31] proposed a human activity recognition system that collected data from an off-the-shelf smartwatch and used an artificial neural network for classification. To take advantage of richer context information from both devices, some studies carried out activity recognition by fusing smartphone and smartwatch sensors [32,33,34]. Shoaib et al. [9] used motion sensors from a smartphone at the right trouser pocket and a smartwatch at the right wrist to recognize 13 different activities, and the results showed that the combination of these two positions outperformed either of them alone. When there are more than two devices worn on the body, it is necessary to use a wireless body area network to integrate sensing, computing, and wireless communication into a unified framework, as in studies [10,35].

Bulling et al. [36] provided a comprehensive introduction to the standard procedures and best practices for HAR tasks, and claimed that feature selection is needed to minimize memory, computational power, and bandwidth requirements, particularly for real-time processing on embedded systems, for the reason that the dimensionality of the feature space is proportional to the quantity of training data and computational resources. Feature selection solutions can be categorized into three different groups: filter, wrapper, and embedded [37]. Suto et al. [38] presented a conceptually simple naïve Bayesian wrapper feature selection method and compared it with some widely used filter feature selection algorithms, and the results demonstrated that the wrapper technique outperformed filter algorithms in HAR tasks. Many other previous works focus on one of these feature selection solutions to HAR problems [39,40,41,42,43,44,45]. Among these, some typical feature selection schemes are often used in the literature, such as correlation-based feature selection (CFS) [43,46], Relief-F [43,47,48], and sequential forward floating search (SFFS) [48,49]. Also, some other projection-based dimensionality reduction techniques are commonly applied for HAR tasks, like principal component analysis (PCA) [49,50,51] or kernel principal component analysis (*k*PCA) [22]. Nowadays, deep learning techniques have begun to be adopted in place of well-established analysis techniques that rely on hand-crafted feature extraction and selection, and have demonstrated their effectiveness in HAR tasks [23,52,53]. Chen et al. [54] presented a survey of the state-of-the-art deep learning methods for sensor-based HAR and formed an overview of the current research progress. From this study, we can see that although layer-by-layer structures of deep neural networks are good at encoding and selecting features from multiple perspectives, they still face many challenges, such as the interpretability of features, which makes it difficult to understand which part of features facilitates recognition and which part deteriorates that. Moreover, there exist some studies using reinforcement learning for feature selection in HAR tasks. For example, considering the multi-modality feature of sensor-based HAR, Chen et al. [55,56] proposed to use reinforcement learning combining attention mechanisms for activity recognition, respectively. Bhat et al. [57] applied an online neural network classifier with reinforcement learning to recognize common daily activities in real-time while consuming low power. The RL-based methods make the feature selection process more adaptive.

Among existing works on feature selection using the wrapper approach, metaheuristics based on swarm intelligence, such as ant colony optimization (ACO) [58], genetic algorithm (GA) [59], and particle swarm optimization (PSO) [60], have been shown to be very promising approaches. Inspired by the foraging behavior of natural bees, Sadeg et al. [61] proposed a metaheuristic algorithm, BSO-FS, to solve the feature selection problem, and the results showed that it could select efficiently relevant features while improving the classification accuracy for some public datasets. On basis of BSO-FS, a hybrid metaheuristic, QBSO-FS, was presented to make the search process more efficient and adaptive by integrating Q-learning with BSO, which gave very satisfactory results compared to recently published algorithms [62]. However, a Q-learning scheme depends on the stored lookup table of Q-values. Once the state and action space expand and become too big, it will become computationally intractable. As an extension of Q-learning, a deep Q-network has stronger learning ability. In this paper, we take advantage of the merits of BSO and DQN, design a new feature selection scheme for human activity recognition tasks by integrating these two algorithms, and try to improve activity recognition performance.

## 3. Proposed Method

Human activity recognition based on wearable sensor data is essentially a multi-class classification problem. It aims at mapping the readings of wearable sensors to a predefined set of human activities. Since the sensor data is often noisy, it needs to be denoised first. Then, the data stream of wearable sensors can be segmented into small time windows, each of which reflects the current motion context in this time range. To perform activity classification, it is necessary to extract as many features as possible heuristically from the data sequence in a window and form a feature vector. Usually, there exist irrelevant features in the vector, and the dimensionality of the vector is too high for real-time applications on devices with limited resources. Therefore, dimensionality reduction processing is required, which is fulfilled by feature selection operation. Finally, the feature vectors after selection are obtained and taken as the input into a classifier. During the training phase, the feature vectors are used to train the classifier model. In the prediction phase, they are used to recognize unlabeled activities.

**Figure 1:**
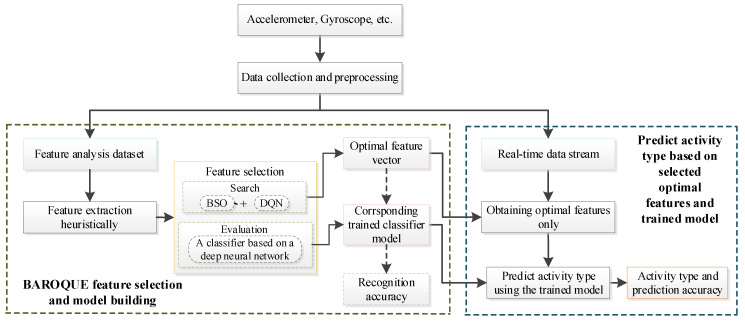
Structure of the proposed scheme for human activity recognition. It is made up of two stages: feature analysis and model building, and real-time human activity prediction.

Among them, feature selection is one of the critical steps to ensure performance and efficiency. In this paper, the schematic diagram of human activity recognition is shown in Figure 1. It consists of two major stages. In the first stage, we employ the proposed hybrid feature selection method to search through the feature space and design a deep neural network model to evaluate the selected features. According to the recognition accuracies obtained using different feature subsets, we can obtain the optimal feature subset and its trained evaluation model. In the second stage, the real-time data stream is obtained and preprocessed, the features in the optimal subset are extracted, and the activity type is predicted based on the corresponding trained model.

### 3.1. Data Collection and Preprocessing

At the beginning of a typical HAR task, raw wearable sensor data is acquired using several sensors attached to different locations on the body. For convenience to be used anytime, anywhere, HAR applications usually employ off-the-shelf commercial devices, such as smartphones and smartwatches. The sensors embedded in these lightweight devices can unobtrusively quantify activities of daily living and provide long-term objective, insightful measures for HAR applications. Among them, accelerometers and gyroscopes are the two most common sensors that are adopted for human activity recognition.

For example, the UCI-HAR dataset [63] was collected by 30 volunteers each performing six activities, namely walking, walking upstairs, walking downstairs, sitting, standing, and laying, when wearing a smartphone on the waist. The WISDM dataset [64] was acquired at a rate of 20 Hz from 51 test subjects wearing a smartphone and smartwatch as they conducted 18 activities for 3 min apiece. For these two datasets, in addition to the raw accelerometer and gyroscope sensor signals, pre-extracted feature sets were also provided, with 561 features for the former and 92 features for each sensor in the latter. UT_complex dataset [9] was collected by 10 participants performing 13 human activities, e.g., walking, jogging, sitting, standing, biking, using stairs, typing, drinking coffee, eating, giving a talk, and smoking, with each carrying two mobile phones, one in the right pocket and the other on their right wrist to emulated one smartwatch. The data were obtained at 50 samples per second from each phone’s accelerometer, linear acceleration sensor, gyroscope, and magnetometer. Similarly, the SMT dataset [35] was obtained by recording sensor data at 50 Hz from the three-axis accelerometer and three-axis gyroscope from both a smartphone and wrist-worn device worn by nine participants when performing 12 daily activities. These datasets all have been used and tested in some previous related works and will be utilized for evaluation and analysis here. For more detailed experimental setup on these datasets, please refer to the relevant references cited therein.

The raw signals from these IMUs are generated over time continuously, and they can be collected at a certain sampling frequency into a long time series. For example, the sensor signals in the UCI-HAR dataset were captured at a constant rate of 50 Hz, which means 50 samples were obtained per second from each sensor channel such as the *X*-axis of the accelerometer. Given the complexity of application scenarios, the collected data may have some errors, like missing values or high-frequency noise, and it should be preprocessed before being put into the subsequent operations. First, the missing values are estimated and replaced according to a certain rule, for example, using the mean of available items. Then, the long sequence is divided into time-series segments of constant or variable length using sliding window techniques. Next, there follows a low-pass filter operation to remove noise. Additionally, some other preprocessing methods can be used here, like data re-sampling, data normalization, and standardization, etc. Taking the UCI-HAR dataset as a case, to obtain the pre-extracted features, the sensor signals were divided into fixed-width sliding windows of 2.56 s and 50% overlap, and a median filter and a 3rd order low pass Butterworth filter with a corner frequency of 20 Hz were used to remove noise.

### 3.2. Feature Extraction

Classification algorithms for human activity recognition usually do not directly operate on the raw data, but rely on various features computed from that data. Feature extraction is known as the conversion of given raw data into a set of features. By operating on the raw data, it expects to generate descriptive, meaningful and non-redundant information, which makes it possible to analyze data from a high-level perspective, simplify the consequent learning process, and improve the recognition performance eventually. Extracting features locally on the mobile devices and transmitting just the results can also reduce the communication load compared with full transmission of the raw data. To ensure good classification performance, all kinds of miscellaneous features should be taken into consideration. The usual practice is to extract as many features as possible heuristically and then select the most relevant ones by feature selection.

The features are calculated over the aforementioned time-series windows of constant length, which are largely overlapping. Let us assume the window size is *W*, for each window of time series, the corresponding data $S_{w}$ needs to be mapped into a well-defined feature space with problem specific dimensionality *D* to form a feature vector $F_{D}=\left(f_{w,1},f_{w,2},\ldots ,f_{w,D}\right)$. Therefore, a feature can be regarded as a function to learn the statistical distribution of data points. Regarding human activity recognition tasks, different patterns will be generated for different activities. For example, the “jogging” activity shows a particular pattern due to the way the user moves his/her body, which is quite different from the “bicycling” activity pattern. The extracted statistical distribution by feature extraction from the raw sensor data provides vital information to distinguish between the patterns for “jogging” and “bicycling”.

Table 1 shows some popular features extracted from three axial data from both an accelerometer and gyroscope for HAR tasks, many of which are commonly used across existing studies of activity recognition [36]. Such features mainly involve three different types, including time, frequency, and time-frequency domain features. Time-domain features are the most popular in related studies, such as mean value, median, variance, skewness, kurtosis, percentiles and interquartile range, and so on. To quantify the similarity between signals coming from different axes, cross-correlation coefficients are also used. To obtain descriptions of the energy and power contained in signals, the Fourier transform is first applied on the time series, and then the statistical features are calculated, such as signal power and spectral entropy.

### 3.3. Feature Selection

In real-world application scenarios, to ensure the best possible representation, it is necessary to introduce as many features as possible due to the absence of a priori knowledge about what features are relevant to predict the activities in the current HAR application. Unfortunately, irrelevant or redundant features can result in classification performance degradation and an unnecessary increase of computational overhead. In the online HAR scenario using a mobile phone in particular, it may become an impossible task. Therefore, choosing relevant features among a large initial set to generate a representation subset often allows delivering better recognition results. Furthermore, it brings some other advantages, for example, making data visualization and data understanding easy, avoiding overfitting issues during training, reducing training and prediction time, and addressing the curse-of-dimensionality issue.

Generally, feature selection schemes are grouped into three categories: filter methods, wrapper methods, and embedded methods [37]. Simply put, a feature selection method falls into one of the three categories according to whether it uses a learning algorithm or not and the way it uses it. In the filter approach, the algorithm evaluates the usefulness of features according to heuristics based on general characteristics of the data without involving a learning procedure. By contrast, the wrapper and embedded methods are different in both using a modeling algorithm. Among these two kinds of method, the former considers feature subsets by their quality of the performance on the modeling algorithm, while the latter performs feature selection during the modeling algorithms execution. The proposed feature selection scheme, BAROQUE, follows the wrapper approach, which combines a bee swarm optimization metaheuristic with a multi-agent deep Q-network to select the most relevant feature subset and evaluate it using a classifier for HAR tasks.

#### 3.3.1. Bee Swarm Optimization Metaheuristic

In nature, foraging behavior is essential for a hive to survive. To fulfill such a task, in the beginning some bees are sent out to discover food sources and gather nectar. When they come back home and unload the nectar, they dance to share information such as the distance, direction, and quality of the food source with other bees in the hive. Relying on instinct, the other bees figure out the bee with the optimal solution and follow it to get to the best nectar source.

Inspired by the foraging behavior of natural honey bees, researchers present an innovative collective intelligence-based scheme, the BSO metaheuristic, to solve optimization problems. It provides the capability of self-organization and self-adaptation to the environment and dynamic task assignment. Based on this idea, some BSO-related algorithms are proposed to deal with different problems in the last few years. These algorithms have been proved to perform better in solving certain numerical optimization problems than some other collective intelligence-based algorithms [61].

Specifically, BSO is devised as an iterative search process to provide solutions to an optimization problem based on a population of cooperating artificial bees. The iterative algorithm mainly includes three steps: determining the search region for each bee based on an initial reference solution, carrying out the local search by each bee and obtaining the best local solutions, and selecting the new reference solution from these best local solutions. To be specific, in the beginning a solution is created as the initial entry point of the search procedure, randomly or via a heuristic. It is going to act as the reference solution, *ref_sol*, from which a set of candidate solutions named *search_region* are determined. Then, each of these solutions is designated to act as the starting point of the local search process of a corresponding bee. For each bee, it carries out a local search procedure and obtains the best solution at the end of the search. Then, it delivers the best-found solution to the other bees via a table called *dance*. From this table, one of the solutions will be selected to be the new reference solution for the next iteration. To avoid getting stuck in cycles, the reference solutions are saved into a *Tabu* list.

There are two important parameters in BSO, i.e., *flip* and *max_chances*. The former is employed to generate the set of solutions that define the search region. It manages to keep each of the solutions in the same distance with *ref_sol* by a distance inversely proportional to *flip*. Therefore, the *flip* parameter should be chosen carefully for the sake of good coverage of the search space. The latter is mainly used by the reference solution selection subprocess to avoid getting stuck in local optima. Based on it, the subprocess maintains a record of the number of remaining chances to hang around the current search region. When the number of attempts reaches *max_chances*, the corresponding bee will escape this search area to exploit another one.

To be specific, during the subprocess of reference solution selection, by implementing judicious intensification and diversification mechanisms, BSO wisely takes into consideration both exploiting the current promising search region and exploring new search regions to achieve good coverage of the search space quickly. It makes a choice between intensification and diversification according to the results obtained in a search region during one search iteration. The new reference solution *ref_sol* for the next iteration will be set to the current best global solution as long as its performance gets improved. Apart from that scenario, if the best local solution performs better than the current *ref_sol*, then an intensification is carried out, and the number of chances minus one. Until the number of chances decreases to 0, the furthest solution among all the solutions stored in the *Tabu* list will be chosen as the new *ref_sol* to perform a diversification. The algorithm will keep running until the predetermined termination goal or the maximum number of iterations is reached. The overall algorithm flow can refer to Algorithm 1 in Section 3.3.5.

#### 3.3.2. BSO for Feature Selection

Some previous related works have applied BSO to feature selection by adapting the general algorithm to specific problem domains. Taking the application scenario of feature selection in HAR tasks as an example, each bee is just like a worker employed to search a local region of the whole feature space, aiming to find the optimal feature subset with other bees collectively. In this paper, we follow the way of BSO on how to encode a solution or determine a search region, as well as the way to evaluate the quality of the selected feature subset for HAR tasks.

Here, we define a solution as a selected feature subset out of the initial feature set. To represent a solution, a binary vector of length N is used, with N standing for the number of all the original features. If the *i*-th entry of the vector is set to 1, this means the corresponding *i*-th feature is selected, otherwise it is set to 0. Moreover, the quality of the solution is represented by fitness and denoted as *f*(*s*). Many commonly used evaluation metrics can act as fitness, such as classification accuracy, precision, and f1-score. It is worth noting that if two solutions have the same fitness, it is wise to consider the one using fewer features, due to the fact that fewer features mean less computation and high efficiency.

Based on the reference solution, a set of solutions are generated, which defines the search region. The number of the solutions equals that of the bees, which is denoted as *nb* here, because each bee will be assigned a solution to act as a starting point of its local search. These *nb* solutions are obtained by flipping some bits in the *ref_sol* vector, and the subscriptions of these bits can be calculated by two parameters, N and *flip*, where *flip* is an empirical value. As the value of *flip* determines the distance between *ref_sol* and the solutions defining the search region, it plays a pivotal role in performance optimization for the search process. If the value of *flip* is too small, BSO tends to favor the exploration instead of exploitation of the search space. If the value is too big, BSO tends to converge to a local optimum. Therefore, an appropriate value should be chosen to achieve a good tradeoff.

Specifically, to obtain the solutions spread as evenly as possible in the search space, three different strategies are used to generate the solutions. In the first strategy, the *i*-th solution is created by flipping the bits in *ref_sol* at a regular interval defined by *flip* and starting at the *i*-th entry. For example, let us assume N = 16, *nb* = 10, and *flip* = 4, and then the first four solutions, $s_{0}$, $s_{1}$, $s_{2}$, and $s_{3}$, will be obtained by flipping the bits with following subscripts: (0,4,8,12), (1,5,9,13), (2,6,10,14), and (3,7,11,15), respectively. In the second one, a number *k* is obtained, and N*/flip* contiguous bits are flipped starting by the *k*-th bit. Therefore, the next four solutions following the previous example, $s_{4}$, $s_{5}$, $s_{6}$, and $s_{7}$, are obtained by flipping the following bits: (0,1,2,3), (4,5,6,7), (8,9,10,11), and (12,13,14,15), respectively. In the third one, the last two solutions, $s_{8}$ and $s_{9}$, are generated randomly according to Pareto’s law.

#### 3.3.3. Deep Q-Network

Deep Q-network is one of the classic deep reinforcement learning methods, which combines two different strategies: deep neural networks and Q-learning, and it is a powerful tool for feature selection. As in typical reinforcement learning tasks, we formulate the feature selection problem through a Markov decision process (MDP) in this work. An MDP is denoted by a 5-tuple 〈S, A, R, T, γ〉, where S is the agent’s state space; A is the agent’s action space; T(s,a,s′)=P(s′|s,a) represents the transition dynamics, which returns the probability that taking action a in state s will result in the state s′; R(s,a,s′) is the reward function, which returns the reward received when transitioning to state s′ after taking action a in state s; and γ is a discount factor, which is a value between 0 and 1 to discount the value of the future rewards. A policy π : S→A is a mapping from states to actions, according to which an agent chooses to take a specific action for each state in the environment. The goal of the agent is to find the policy $\pi^{*}$ that maximizes the expected discounted total reward over the agent’s lifetime.

As a key term related to MDPs, Q-function is the mapping from state-action pairs to real space, which is denoted as $Q^{\pi }\;:S\times A\to \;$R. For a given state-action pair (s,a), $Q^{\pi }$(s,a) defines the expected future discounted reward for taking action a in state s and then following policy π thereafter. The best value function $Q^{*}\left(s,a\right)$ obeys the Bellman equation below, and it can be obtained by calculating the following Q-value recursively:

$$Q^{*}\left(s,a\right)=\sum_{s^{\prime }\in S}T\left(s,a,s^{\prime }\right)\left[R\left(s,a,s^{\prime }\right)+\gamma \underset{a\prime }{\max}Q^{*}\left(s^{\prime },a\prime \right)\right]$$

Then, the optimal policy, $\pi^{*}$, can be easily obtained by greedily selecting the action with the highest Q-value in the current state: $\pi^{*}\left(s\right)=\underset{a\in A}{\arg\max}Q^{*}\left(s,a\right)$. This characteristic has incubated many different learning algorithms that seek to directly estimate $Q^{*}\left(s,a\right)$ and retrieve the optimal policy from it. Among these, one of the most popular and widely used algorithms is Q-learning.

Q-learning is a model-free reinforcement learning algorithm to learn a policy, telling an agent what action to take under what circumstances, by gradually improving the quality of Q-values. Before learning begins, Q-value for an agent is initialized to a possibly arbitrary fixed value. Then, its estimate will get improved by iteratively carrying out the steps: taking an action in the environment, observing the reward and next state, and updating its Q-function estimate. Specifically, at each time t the agent selects an action $a_{t}$, observes a reward $r_{t}$, enters a new state $s_{t+1}$ depending on both the previous state $s_{t}$ and the selected action $a_{t}$, and then the Q-value is updated by using the weighted average of the old value and the new information according to:

$$Q^{new}\left(s_{t},a_{t}\right)\overset{\;\;update\;\;}{\leftarrow }Q\left(s_{t},a_{t}\right)+\alpha \cdot \left[r_{t+1}+\gamma \cdot \underset{a\prime }{max}Q\left(s_{t+1},a\prime \right)-Q\left(s_{t},a_{t}\right)\right]$$

where α∈(0, 1]  is a parameter, called learning rate, used for step size smoothing, and $r_{t+1}$ represents the reward received when the state transitions from $s_{t}$ to $s_{t+1}$. Note that, $Q^{new}\left(s_{t},a_{t}\right)$ equals the sum of three parts: $\left(1-\alpha \right)\cdot Q\left(s_{t},a_{t}\right)$ is the original Q-value weighted by the learning rate, which means the held portion of the original Q-value; $\alpha \cdot r_{t+1}$ is the reward weighted by the learning rate, which can be obtained by taking action $a_{t}$ when in state $s_{t}$; $\alpha \cdot \gamma \cdot \underset{a\prime }{\max}Q\left(s_{t+1},a\prime \right)$ stands for the maximum reward that can be obtained from state $s_{t+1}$ by taking the most appropriate action, which is weighted by the learning rate and discount factor.

Implementing classic Q-learning depends on the stored lookup table of Q-values, which caches the Q-values for all possible action-state pairs (s,a). It is guaranteed to converge to an optimal and stable joint strategy as long as it meets some conditions. For example, each Q-value is associated with one unique state–action pair, and the agent visits each state and action infinitely often. When the state and action space expand and become too big, it will become computationally intractable. Moreover, when there are too many states, most of them will not have the chance to be accessed frequently. Therefore, Q-learning performs well for small-scale tasks rather than large-scale tasks.

To address such issues, the Q-value estimate in Q-learning is often implemented with function approximation instead of a tabular function, which allows generalization of experience. To avoid inappropriate function approximation causing divergence, deep Q-learning (DQN) is introduced. DQN is a variation of the classic Q-Learning algorithm. It approximates the Q-function values with a deep neural network that outputs a set of action values Q(s,·;θ) for a given state *s*, where θ are the parameters of the network. There are two primary contributions of DQN that make this work. First, it uses a separate target network, which is copied every few steps from the regular network, to estimate the Q-values of the next state, so that the Q-function estimation is more stable. Second, the agent adds all of its experiences to a replay buffer, which is then sampled uniformly to build mini-batches of training data and update the network based on it.

Specifically, DQN caches a history of the most recent experiences with each of them being a 5-tuple $\left(s,a,r,s^{\prime },T\right)$. The 5-tuple means an agent taking action a in state s, transitioning to state $s^{\prime }$ and receiving reward  r, with T being a boolean indicating whether $s^{\prime }$ is a terminal state. After each step in the environment, the 5-tuple is appended to the experience list. After some predefined number of steps, a mini-batch of samples are randomly selected from the list, on the basis of which the parameters of the Q-network are updated. This process of reusing previous experiences to update a Q-function is also called experience replay. It is typically used in reinforcement learning to accelerate the backup of rewards. The practice of taking fully random samples in a mini-batch from history to update the Q-network avoids bias in the function approximation estimate and decorrelate the samples from the environment. Furthermore, it is useful to estimate the Q-value for the next state using stale network parameters in an experience and updating the network parameters every few steps. By doing so, it provides a stable training target for the network function to fit and gives it reasonable time to do so, which keeps the errors in the estimation under control.

#### 3.3.4. Multi-Agent DQN for Feature Selection

Let us consider the scenario that there exist N features in the initial feature set. To be intuitive, we treat each feature in the set as one agent u∈{0,1,…,N}. Then, the observation of the whole environment at time slot t for agent u is denoted as $o_{u}\left(t\right)$, which is a list of binary values with a length of 2N. The former N entries in the list denote all the features being selected, while the remaining N entries represent the one-hot encoding of the partial observation for agent u. If the u-th feature is selected, then the u-th entry of the partial observation will be set to 1, with the other entries being set to 0. Otherwise, all the N entries will be set to 0.

Accordingly, we denote the action of agent u at time slot t as $a_{u}\left(t\right)$. The action space for every agent is $a_{u}\left(t\right)\in \left\{0,1\right\}$. When $a_{u}\left(t\right)=0$, it represents the *u*-th feature not being flipped, Otherwise, it is flipped. It is worth noting that there is no competition among the agents, which means selecting a feature does not affect the selection of other features. So, the Markov chains for the N features are mutually independent. After agent *u* takes the action $a_{u}\left(t-1\right)\;$at time t−1, whether the corresponding feature entry is flipped or not, it will receive a reward denoted by $r_{u}\left(t\right)$. Here, we design a reward mechanism for each user as follows: Assuming that we take classification accuracy as the evaluation metric, if the classification accuracy based on the selected features at time t−1 (denoted as $acc_{t-1}$) is lower than that at time t (denoted as $acc_{t}$), i.e., $acc_{t-1}$ < $acc_{t}$, then $r_{u}\left(t\right)$ is set to the value of $acc_{t}$; If $acc_{t-1}$ > $acc_{t}$, then $r_{u}\left(t\right)$ is set to the value of 0.5×($acc_{t}-acc_{t-1}$), which is a negative decimal to discourage such action. Additionally, when $\mathrm{the}\;\mathrm{values}\;\mathrm{of}\;acc_{t-1}$ and $acc_{t}$ are equal, $r_{u}\left(t\right)\;$is set to the product of $acc_{t}$ and a design factor, whose sign is determined by the number of the selected features. When the number of selected features at time t−1 is bigger than that at time t, the factor is a positive decimal. Otherwise, the factor is negative. In this way, the subset with fewer features is encouraged to be selected as the solution. After many iterative simulation steps, a set of the previous observations and actions up to time t−1, $H_{u}\left(t-1\right)$ = $\left\{o_{u}\left(k\right),a_{u}\left(k\right)|k=\left(1,\ldots ,t-1\right)\right\}$, are obtained. The learning objective is to find an optimal policy $\pi^{*}$ to maximize the cumulative discounted reward denoted by $R=\overset{U}{\underset{u=1}{\sum }}\overset{T}{\underset{t=1}{\sum }}r_{u}\left(t\right)$ from the above interaction history. During the learning process, a neural network is needed to choose actions and estimate the Q-value associated with the selected actions, and we design the network with the following architecture.

The first component of the DQN is the input layer $I_{u}\left(t\right)$, which is a neuron vector of size 2N corresponding to each observation $o_{u}\left(t\right)$ at time t. Next follows one fully connected (FC) layer with hid_num hidden nodes and using ReLU (rectified linear units) as its activation function. ReLU is a non-linear activation function that can be formulated as max(x,0), which has the advantage of running fast and avoiding vanishing gradient problems. This hidden layer has full connections to all neurons in the input layer, and it is capable of learning non-linear combinations of such neurons and mapping them to a vector of size hid_num. Then, the vector is connected to the output layer $Q_{u}\left(t\right)$, a linear FC layer with a specific number of neurons, and the number of its neurons equals the number of available actions, i.e., 2. Each of the two neurons represents the Q-value for agent *u* taking actions to flip the u-th entry in the feature vector or not at time t, respectively.

#### 3.3.5. BAROQUE: The Proposed Hybrid Scheme

By integrating a multi-agent DQN with BSO, BAROQUE is proposed as a hybrid feature selection scheme for HAR tasks. It has two main functions: search and evaluation.

In BAROQUE, to fulfill the search for optimal feature subset, BSO is used to guide the search process and determine the search region for all bees, while a multi-agent DQN is adopted to improve each bee’s local search. In original BSO, after the solutions defining the search region are generated and assigned to all bees, each bee carries out the local search in the neighborhood of its allocated solution heuristically and finds out the best local solutions by evaluation. Instead, BAROQUE uses a multi-agent DQN to implement the local search for each bee. In this way, all bees can learn from their own experience, which will guide the search process to converge more quickly. Note that, a solution in BSO is encoded as a binary vector of length N with ‘1’s indicating the selected features, while a state in the multi-agent DQN is a vector of length 2N, so there exists a process of conversion. Assuming N = 5 and taking solution (10110) as an example, the current state for the 3rd agent is (0010010110). To narrow down the search space, when the agents choose actions to transition from present to next states for each bee, the resultant states are restricted to the neighborhood of *best_global_sol* as mentioned in Algorithm 1. In the case of *solution* = (10110) and *best_global_sol =* (01101), the 1st, 2nd, 4th, and 5th bits of these two are different, and we can easily obtain this conclusion by XOR operation. Therefore, when the agents choose actions, only the operations of flipping these bits are accepted.
**Algorithm 1.** Overall algorithm flow for BSO and BAROQUE.**Input:** An instance of a combinatorial optimization problem **Output:** The best solution found 1: Initialize a reference solution, *ref_sol*, at random or via a heuristic 2: **while** not stopping criterion **do** 3:  Determine *search_region* from *ref_sol* 4:  Set the value of *n_chances* as *max_chances* 5:  Assign a solution from *search_region* to each bee 6:  **for** each bee *b*
**do** 7:   Carry out a local search 8:   Store the result in the *dance* table 9:  **end for** 10:  **if** *best_sol* is better than *best_global_sol*
**then** 11:   Set the value of *best_global_sol* as *best_sol* 12:   Set the value of *n_chances* as *max_chances* 13:   Intensification 14:  **else** 15:   **if** *n_chances* > 0 **then** 16:    *n_chances* minus one 17:    Intensification 18:   **else** 19:    Diversification 20:   **end if** 21:  **end if** 22: **end while** 23: **return** *best_global_sol*

Step by step, by taking turns to carry out search and evaluation operations iteratively in BAROQUE, the best local solutions are discovered, the best global solutions are updated, and the corresponding optimal feature subsets will eventually reveal themselves.

### 3.4. Evaluation and Prediction

In human activity recognition, a classifier is needed for both major stages as shown in Figure 1. In the first stage, the classifier is used to evaluate the quality of solutions during the feature selection and model building process. In the second stage, the classifier is used to carry out real-time activity prediction during an application process.

As for the evaluation and prediction, any algorithm capable of resolving non-linear classification problems will theoretically work, like *k*NN and SVM. Here, we also design a multi-layer neural network to act as the classifier to improve the classification accuracy. The input layer of the neural network consists of a number of neurons, the number of which is equal to that of selected features. The output of the neural network represents the classification probabilities for all the available activity types, so the corresponding neurons and the activity types share the same number. Between them, there are two convolutional blocks and three fully connected (FC) layers. Each of the convolutional blocks is made up of three parts: a convolution layer, a rectified linear unit (ReLU) activation function, and a max pooling layer. Due to the inertial sensor data being one-dimensional time-series, we set the kernel size for these two convolutional blocks to 5 × 1 and 3 × 1, and use 32 filters and 64 filters in them, respectively. As an elementwise activation function thresholding at zero, ReLU outputs the input directly if it is positive, otherwise, it outputs zero. It is non-linear and leaves the size of the layer unchanged. Each max pooling layer performs a down-sampling operation with a kernel size of 1 × 2 and a stride of 2. The convolutional blocks are followed by three FC layers, and the output of these prior layers is flattened and taken as the input into the FC layers. The number of neurons in the first FC layer equals that of the flattened output of the prior layers. We set the number of neurons in the following two FC layers to 256 and 64, respectively. After layer-by-layer learning, the classifier can map the selected features into activity types and obtain the classification probability for each type.

To carry our evaluation and prediction, the classification metric, *accuracy*, is adopted here. It can be calculated by the formula below based on the chosen classifier:

$$accuracy=\frac{TP+TN}{TP+FP+TN+FN}$$

where *TP*, *FP*, *TN*, and *FN* represent the number of true positives, false positives, true negatives, and false negatives, respectively. Based on this, the fitness in BSO and rewards in DQN can be calculated.

As the main part of the learning process, training the multi-agent deep Q-network and the neural network classifier is a computationally intensive operation, which is usually carried out offline on a powerful server or platform commonly with GPU resources. After learning, the obtained optimal feature subset can be extracted directly on wearable devices like smartwatches or smartphones, and the corresponding models can be used to predict the real-time human activity on mobile devices like smartphones.

## 4. Experimental Results and Analysis

To evaluate the proposed hybrid algorithm BAROQUE on feature selection for HAR tasks based on wearable sensors, we implemented it as well as some previous related studies in Python and used the results for analysis. Here, Pytorch was used to build neural network models for DQN and the evaluation classifier, and Scikit-learn was also used to provide auxiliary utilities, such as classification algorithm implementation of kNN and SVM, data-processing libraries, and evaluation metrics. Experiments were conducted on a PC running Windows 10 system and equipped with an Intel Core i7-7740x CPU @ 4.30 GHz, 32 GB of RAM memory, and an NVIDIA GeForce GTX 1080 Ti. The available GPU can be used to speed up the training process in some experiments. In this section, four datasets were utilized for evaluation and analysis, i.e., the UCI-HAR, WISDM, UT_complex, and SMT datasets. Among these datasets, the first two datasets were downloaded from the UCI machine learning repository. One dataset was from a research program working on complex human activity recognition. The other one was collected for our previous related study. Based on them, we first analyzed the raw inertial signals for HAR tasks and explored the impact of feature selection by BAROQUE. Then, we compared the proposed hybrid approach with some existing related methods and performed a detailed analysis in terms of accuracy and efficiency.

### 4.1. Analysis of Raw Signals

**Figure 2:**
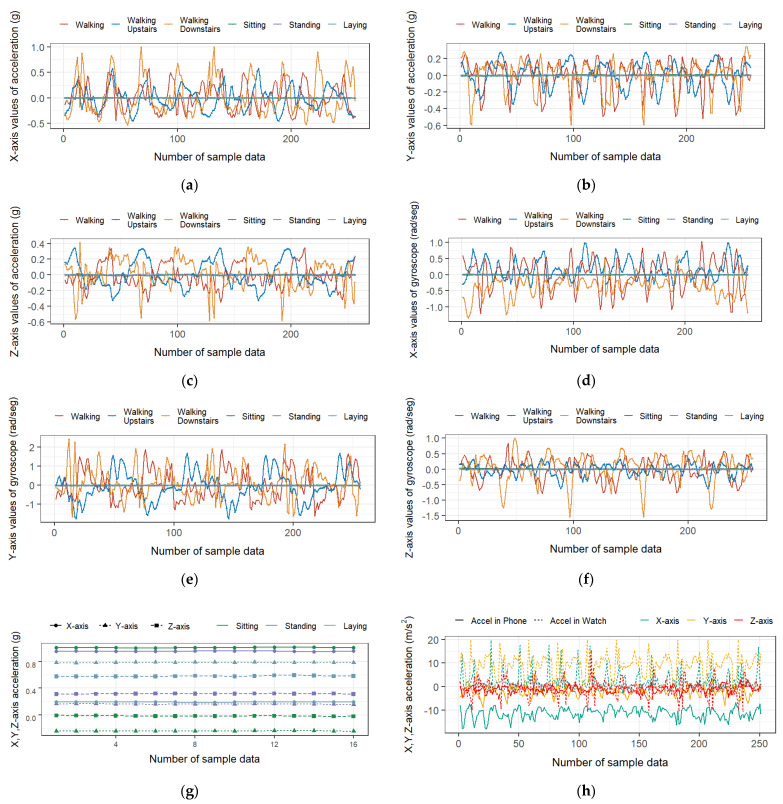
Data samples from accelerometers and gyroscopes. (**a**–**f**) The *X*, *Y* and *Z*-axis linear acceleration data and angular rate data in the UCI-HAR dataset; (**g**) the *X*, *Y* and *Z*-axis values of total acceleration including gravity when performing sitting, standing and laying in the UCI-HAR dataset; (**h**) the *X*, *Y* and *Z*-axis values of acceleration both from the smartphone and smartwatch when performing walking in the WISDM dataset.

First, let us take a look at the raw signal data in these datasets. Figure 2a–f show the *X*, *Y* and *Z*-axis values of linear acceleration and the *X*, *Y* and *Z*-axis values of angular velocity in the UCI-HAR dataset, which can be seen as the raw features for activity recognition. From them we can see that there are some patterns in the raw inertial signals for the three kinds of activities including walking, walking upstairs and walking downstairs. However, for the activities of sitting, standing and laying, it is intractable for the raw features to distinguish from each other since these are stationary activities and there exists no relative acceleration at all. Figure 2g shows the *X*, *Y* and *Z*-axis values of total acceleration including gravity, from which we can see that the three activities of sitting, standing and laying can be distinguished from each other due to the sensors’ different orientation relative to gravity. Therefore, we can know that one extra kind of feature may bring about additional information about the performing activities and it is necessary to introduce as many features as possible to ensure a better performance.

Figure 2h shows the *X*, *Y* and *Z*-axis values of acceleration from the smartphone and smartwatch when performing walking in the WISDM dataset. The solid lines stand for the raw signal readings from the accelerometer in the smartphone and the dotted lines stand for those data in the smartwatch, while different colors indicate different axes. From the figure, we can see that the same kinds of sensor mounted in different parts of the human body convey different motion contexts and it is reasonable to select different features for the data from different sensors.

### 4.2. Analysis for Feature Selection

**Figure 3:**
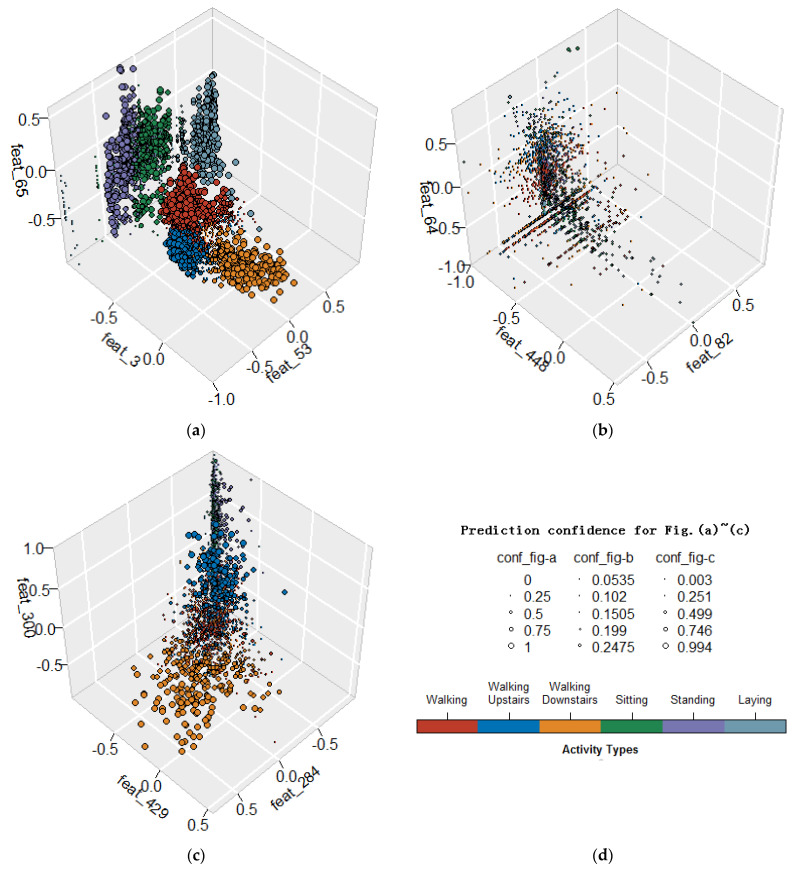
The prediction results for test data using the constructed 3 subsets with each having a capacity of three features from the pre-extracted 561 features in the UCI-HAR dataset. (**a**–**c**) Schematic illustrations of the recognition results for test data consisting of feature subset *T*3, *W*3, and *R*3, respectively; (**d**) two legends of the above illustrations, one using point sizes to distinguish different confidence levels, the other using different colors to represent different activity types.

To validate the impact of feature selection, we first constructed three feature subsets with a capacity of three for the convenience of visualization from the pre-extracted 561 features in the UCI-HAR dataset. Let us denote the original set as O and denote the feature subset obtained by BAROQUE as B, then the subset of unselected features corresponding to the difference of the above two sets was denoted as U=O−B. The constructed feature subsets included the top three features selected from B, the worst three features selected from U, and the three features randomly selected from O. They were represented as *T*3 (including the No. 3, 53, and 65 features in the pre-extracted 561 feature set), *W*3 (including the No. 64, 82, and 448 features) and *R*3 (including the No. 284, 300, and 429 features), respectively. Then, we split the UCI-HAR dataset into two parts with 70% as training data and 30% as test data. In order to verify the performance of the different feature subsets, an SVM model was trained using Scikit-learn package in Python based on the training data for each of the feature subsets, and then the prediction results and error rates for the test data were obtained on the basis of the trained model and shown in Figure 3 and Table 2, respectively.

We use six different colors to mark data samples from six different activity types and use different point sizes to stand for different prediction confidence as shown in Figure 3d. Visually, from Figure 3a–c we can see that *T*3 demonstrates the strongest representation ability, *W*3 has the worst representation ability, and *R*3 falls in between these two extremes. Numerically, this is supported by the error rates in Table 2. More specifically, in Figure 3a, the test data space is divided into several almost disjoint regions, the confidence for one sample being predicted as the ground truth annotation is high, and the error rate for each activity is low. In Figure 3b, all the data samples from different categories mix together chaotically, the corresponding confidence for one sample being predicted to fall in its actual category is unacceptably low, and the error rate for each activity is high, which will lead to poor human activity recognition performance. In Figure 3c, some data samples are correctly predicted with considerable confidence and some others are not. The above experiments tell us a truth that all the features are not contributing equally to aid the recognition process. Due to there being no prior knowledge when confronted with a HAR task, the usual practice is to extract a series of features heuristically and manually and then select the most relevant ones.

Next, we conducted several experiments based on the WISDM dataset. At first, we converted the provided ARFF files containing pre-extracted features into CSV files and replaced all missing values in each file using the mean of the corresponding feature. Then, the data subset containing eight types of activities, namely clapping, writing, eating soup, climbing stairs, folding clothes, playing catch, dribbling a basketball, and kicking a soccer ball, was selected, and the BAROQUE feature selection process was conducted based on the smartphone sensor data and the smartwatch sensor data, respectively. The number of bees, *flip* and *max_chances* were set to 10, 5, and 3, respectively, which were also the default settings for BAROQUE for the following experiments. The obtained feature subset for the smartphone was denoted as *sfp* and the subset for the smartwatch was denoted as *sfw*, and they were used to evaluate the performance for both the smartphone sensor data and the smartwatch sensor data. After getting the dataset ready by splitting it randomly into 90% training and 10% test data for 10-fold cross-validation, based on the dataset, the evaluation was carried out using a *k*NN classifier with the parameter for neighbor number set to 2. From the results in Table 3, we can see that the obtained *sfp* has 83 selected features and *sfw* has 61 selected features. There exist only 28 common features, less than half of the features in *sfp* or *sfw*. When applying *sfw* to test the smartwatch sensor data, we got an accuracy of 95.35%; While applying *sfw* to test the smartphone sensor data, a poor result of 86.01% was obtained. And *sfp* has the same characteristics. This conveys the idea that different sensors, or even the same kinds of sensor worn on different parts of the body, need different feature subsets to ensure a better performance. The proposed BAROQUE can select the suitable feature subset for different sensor data.

### 4.3. Analysis on Hybrid Combination

As two independent kinds of method, bee swarm optimization and reinforcement learning can be used to carry out feature selection on their own, and some previous studies did just that. Based on them, Sadeg et al. [62] presented an algorithm, QBSO-FS, by integrating Q-learning, a reinforcement learning algorithm, with bee swarm optimization metaheuristic to solve feature selection problems. BAROQUE is a hybrid version of BSO with a deep Q-network for generating feature subsets, and it is an improvement of QBSO-FS. To observe the effect of the hybrid combination of the BSO and DQN, BAROQUE was compared with its components, BSO and DQN, as well as QBSO-FS in terms of performance and efficiency.

**Figure 4:**
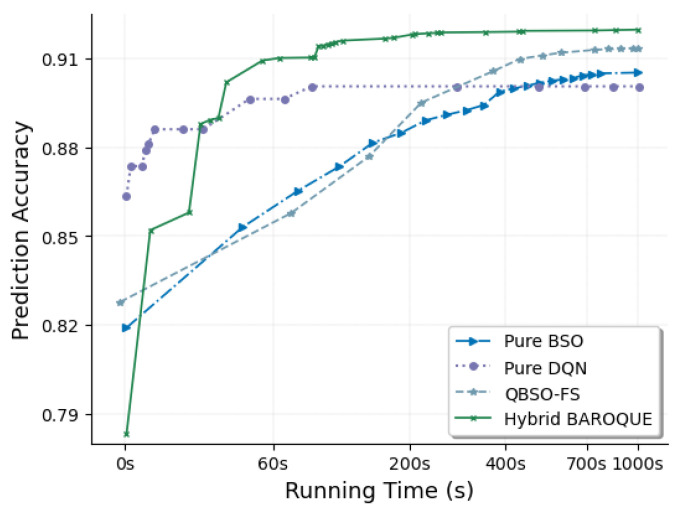
The prediction accuracies of four algorithms, BSO, DQN, BAROQUE, and QBSO-FS, over time based on the 102 features extracted from the three-axis accelerometer and three-axis gyroscope sensor data in the evaluation set from the SMT dataset.

To set up the experiments, the SMT dataset was used here. We first selected a subset of the data from the three-axis accelerometer and gyroscope in the wrist-worn device as the evaluation dataset, which involved the following nine activities, i.e., walking, jogging, bicycling, walking upstairs, walking downstairs, typing, writing, eating, and drinking. Then, 102 features in total were extracted from the three-axis acceleration and three-axis gyroscope sensor signals in the evaluation dataset with a window size of 10 s and an overlap of 8 s, including average, variation, and short-term power spectrum for each channel of data, and the cosine distances and correlation between every two channels of the three-axis data from either of the two sensors. Finally, the obtained feature set was split into training (80%) and test (20%) subsets for evaluation. Based on the training subset, we ran each of the four algorithms, BSO, DQN, QBSO-FS, and BAROQUE, to obtain the relevant features, and evaluated them using these features on the test subset. It is worth noting that the BSO or DQN component in BAROQUE algorithm has the same parameter settings with the corresponding standalone BSO algorithm or DQN algorithm, such as the same learning rate, the same *flip* value, the same *max_chances* value, and the same ε as well as its decay value. And this property still holds when in QBSO-FS algorithm. During each search process, we recorded the changing prediction accuracies over time. For each algorithm, we repeated the process four times and obtained the final results by calculating their average values. Moreover, we drew a curve of the prediction performance over time as shown in Figure 4, in which every turning point means a change in prediction performance.

From Figure 4 we can see that there is an upward trend in prediction performance over time for all four algorithms. Generally speaking, in the beginning, a relatively lower accuracy is obtained for each of the algorithms due to the randomly heuristic feature selection initialization. Then, in the first few iterations, the performance improved quickly. During the following many iterations, the speed of performance improvement gradually slows down. Eventually, the accuracy curve converges smoothly to a stable high value. To be specific, among the four algorithms, the BSO algorithm has the advantage of keeping a steady and smooth growth in accuracy, while DQN algorithm is capable of achieving a good performance very quickly. However, from another perspective, the former takes a long time to obtain a good performance, and the latter tends to get trapped in local optima after the first few training steps. As a hybrid approach by integrating BSO with DQN, BAROQUE can leverage the merits of these two algorithms, reach a high prediction accuracy quickly in the first few iterative steps, and then keep steady growth until converging to a better result. As for the QBSO-FS algorithm, it takes more time to achieve a certain accuracy than BSO algorithm at the beginning, but after some training steps, the performance is improved greatly and reaches a better performance than either BSO or DQN. From Figure 4, we can also see that in a certain time range BAROQUE achieves the best performance among the four algorithms.

### 4.4. Comparison with Other Feature Selection Methods

One of the main purposes of feature selection is dimension reduction in a large multi-dimensional data set, which is essential when the number of features is large. There are some classic dimension reduction methods in the field of machine learning, like PCA and *k*PCA, and they have been widely used in HAR tasks [22,49,50,51]. Some other feature selection algorithms can also be often found in HAR-related research works, like CFS, Relief-F, and SFFS. Here, we carry out feature selection on the UT_complex dataset using each of them and compare our BAROQUE with these methods in terms of prediction accuracy.

PCA is an exploratory data analysis tool for reducing the dimensionality of large datasets, increasing interpretability but at the same time minimizing information loss. It does so by computing the principal uncorrelated components and using them to perform a change of basis on the data, which can successively maximize the variance. *k*PCA is an extension of PCA with kernel techniques. It enables the originally linear operations in PCA to be performed in a reproducing kernel Hilbert space by using a kernel. Relief-F is a commonly used filter method, and it uses a statistical approach rather than a heuristic search to rank the features and prune insignificant features. The algorithm assigns a relevant weight to each of the potential features and selects the ones above the preset threshold. Feature relevance is based on the ability for instances from different classes and instances from the same class to be distinguished. Instead of providing a subset of features, Relief-F weights all features according to relevance. Therefore, an appropriate number of features to include in each subset needs to be determined by processing the ranked feature list with a classifier. CFS evaluates the relevance of features from a correlation-based heuristic, which examines inter-correlation among features as well as their ability to predict classes. Since the features are expected to correlate with each other, it is necessary to identify features that can be used together to increase performance, without being redundant. Therefore, CFS selects features that are highly correlated with the class and uncorrelated with each other. SFFS is a greedy algorithm for finding the most discriminative features, and it is computationally costly. It adds features one at a time to the selected feature subset, and feature selection is performed sequentially in SFFS. To be specific, SFFS is first used to produce a series of feature subsets, and then a discriminatory feature subset candidate is determined among them using a classifier.

To prepare for the experiments, 648 features were extracted from the sensor data in the UT_complex dataset. Specifically, 26 features were extracted from each of the 12 data channels, which are three-axis acceleration, three-axis linear acceleration, three-axis gyroscope data, and three-axis magnetic data. The features consist of two main parts, the time-domain part including mean, variance, standard deviation, mode, median, max, min, zero crossing rate, interquartile range, skewness, and kurtosis for the raw signals, and the frequency-domain part including DC component, the top three frequencies, and the four features for shape and amplitude of the spectra, i.e., mean, standard deviation, skewness, and kurtosis, respectively. Additionally, the Pearson product-moment correlation coefficients for every two channels of the three-axis data from each of the four sensors were also extracted. On basis of the feature set, we carried out feature selection using PCA, *k*PCA (with a linear kernel), CFS, Relief-F, SFFS, and BAROQUE, respectively, and the results are shown in Table 4. To make the evaluation more efficient, we split the training data into 10 parts with one of them as the test set, and used a *k*NN classifier with the parameter for neighbor number set to 5 for each scheme.

After a certain number of iterations for BAROQUE learning, we obtained 134 relevant features. Then, we configured the other algorithms to let each of them output 134 features. From Table 4 we can see that BAROQUE achieves a better performance than other methods except for SFFS, although the differences among these methods are not obvious, and BAROQUE consumes less learning time than other methods except for PCA. Furthermore, we can find that PCA needs the least computing time, and *k*PCA takes a little more time for the usage of a kernel. However, when performing online prediction once the selection process has finished, these two algorithms have to extract all features and transform them into a lower dimension, while other schemes only need to extract the selected features. We find that the *k*PCA using a *linear* kernel achieves a better accuracy than those with a *sigmoid*, *poly*, or *rbf* kernel here. Moreover, SFFS performs closest to the optimal solution, but it takes much longer during the feature selection phase. It is worth noting that Relief-F takes acceptable computing time and achieves a satisfactory accuracy, which makes it a good alternative feature selection scheme. By contrast, CFS consumes a considerable amount of computing time and obtains a much worse accuracy than the others.

### 4.5. Comparison with Other Swarm-Based Methods

As a swarm-based algorithm, we compare the results of BAROQUE with other popular swarm-based algorithms to evaluate its performance. Nowadays, genetic algorithm (GA), ant colony optimization (ACO), particle swarm optimization (PSO) and other algorithms based on swarm intelligence have been applied to select features for HAR tasks [65,66,67]. Table 5 presents the results of No-FS (i.e., without feature selection at all), GA, Binary PSO (BPSO), ACO, and BAROQUE for the previously mentioned datasets.

As for the datasets, we use the 561 features for the UCI-HAR dataset as explained in Section 4.2, and we use the 648 features for the UT_complex dataset as explained in Section 4.4. Moreover, there are two feature sets for WISDM, which are based on the smartphone or smartwatch sensor data, respectively. We name them as well as the fused dataset as WISDM_P, WISDM_W, and WISDM_A, and use them in the experiments.

To facilitate performance comparison, we implemented GA, BPSO, ACO algorithms in Python, and an SVM classification algorithm with *gamma* = 0.001 from Scikit-learn was adopted as the classifier. To set up the experiments, we configured the algorithms using the following settings: the threshold value, crossover rate, and mutation rate for GA were set to 0.5, 0.8, and 0.01, respectively; the threshold value, inertia weight, and two acceleration factors for BPSO were set to 0.5, 0.9, 2, and 2, respectively; the number of ants, initial pheromone amount per path, amount of pheromone per update, and evaporation rate for ACO were set to 10, 1, 0.1, and 0.95, respectively; and BAROQUE used the predefined default parameters. For one experiment, each of the datasets was split into 90% training data and 10% test data randomly, and based on them each of the schemes ran iteratively until convergence. This process was repeated four times and the averaged accuracy is listed as the results in Table 5.

From Table 5 we can see that, each of the four schemes can achieve an acceptable accuracy for activity recognition. Among them, BAROQUE is a competitive algorithm and usually outperforms the other feature selection algorithms for the datasets used in the experiments under most circumstances. It is worth noting that the datasets with all features do not always achieve the best performance, although they usually perform much better than the subsets by most of the feature selection algorithms. During the experimental process we also find that, given enough training time and number of iterations, all the feature selection schemes have a certain probability to obtain a much better accuracy, and even achieve the optimal solution.

### 4.6. Comparison with Other HAR Solutions

Based on wearable sensor data from a smartphone or/and a smartwatch, some researchers have proposed many solutions for human activity recognition tasks. Some of them tested their methods using the above benchmark dataset. These methods adopted different feature selection algorithms and achieved a good recognition accuracy by different classification algorithms, respectively. Here, we conduct an analysis on the performance of such methods as well as our BAROQUE.

Based on the raw data in the UCI-HAR dataset, there are four previous related studies as follows. Here, we compare these four studies with the proposed BAROQUE in terms of classification accuracy, and the results are shown in Table 6.

Anguita et al. [63] first processed the raw data and then trained a classification model using multiclass SVM (MC-SVM). A total of 561 basic features, like frequency skewness, angle between vectors, energy of different frequency bands were extracted from the raw sensor signals in UCI-HAR, and then the feature set was split arbitrarily into a training subset (70%) and test subset (30%). On basis of this, the model was evaluated using a one-vs.-all multiclass SVM with a Gaussian kernel, and a 96% classification accuracy was claimed.

Ronao et al. [23] designed a convolution neural network (Convnet) and trained it on the UCI-HAR dataset for HAR tasks. The neural network made up of four alternating convolutional layers and max pooling layers is capable of extracting basic features in the lower layers and complex features in the higher layers from the raw inertial sensor data. During the training process, the settings for number of layers, number of feature maps, filter sizes, and pooling sizes, were obtained in a greedy search way. The best outcome obtained by them was around 95.75%, which proved Convnet is a good model for activity recognition although considerable resource consumption is needed for training.

Myo et al. [68] presented a new feature selection method, named the cyclic attribution technique (CAT), to assist in recognizing human activities based on group theory and fundamental properties of the cyclic group with a binary operation involving some special properties. An artificial neural network with two hidden layers using a feed-forward propagation algorithm was adopted as the classifier, and it was trained on the UCI-HAR dataset with 70% for training and 30% for testing. This method was claimed to obtain the most important features by removing 498 features from 561 and get a better accuracy of 96.7%.

Ahmed et al. [69] presented a hybrid feature selection method including a filter and wrapper method. They first extracted and selected desired features using a sequential floating forward search, and then fed them to a multi-class support vector machine (SMC-SVM) with non-linear kernel tricks. The model was validated using the UCI-HAR dataset, which produced a result of 98.13%.

In a similar fashion, the proposed hybrid feature selection scheme, BAROQUE with the designed neural network as the classifier, was implemented and ran on the 561 features in the UCI-HAR dataset with a training–test ratio of 7:3. After the model was trained, we obtained the prediction accuracy on the test data. It showed that the proposed model achieved a 98.41% accuracy, which is better than the others.

## 5. Conclusions

This study presented a hybrid feature selection scheme, BAROQUE, by integrating a bee swarm optimization metaheuristic with a multi-agent deep Q-network, which improved the performance and efficiency for human activity recognition based on wearable sensors. Before recognizing human activities, the conventional practice is to extract as many features as possible in advance. However, not every feature contributes to activity recognition equally; rather, too many irrelevant features would degrade the performance and efficiency of the classifier. Thus, BAROQUE has been proposed to play an important role in selecting the relevant features by using a hybrid BSO with a multi-agent deep neural network to search through the feature space in its subset generation step. The experimental results showed that for the benchmark datasets, the selected feature subset obtained by reducing the redundant features could achieve better classification accuracy than other randomly selected feature subsets. Comparison between BAROQUE, BSO, DQN, and QBSO-FS showed that BAROQUE integrated the advantages of both BSO and DQN, and achieved the best performance among these four schemes. In terms of execution time, the experimental results showed that BAROQUE achieved a better classification accuracy using less time and maintained steady growth over the learning time. Finally, the comparison of the results of BAROQUE with those of previous related feature selection algorithms showed that our scheme obtained relatively satisfactory results, and the proposed human activity recognition model outperformed other state-of-the-art HAR models. In the future, we will test BAROQUE on some datasets from more challenging, realistic application scenarios and improve it accordingly.

## Figures and Tables

**Table 1:** Common features extracted from inertial sensor data.

Extracted Features	Equation	Extracted Features	Equation
Mean	$\mu =\frac{1}{N}\sum_{i=1}^{N}x_{i}$	Cross Correlation	$\rho_{i,j}=X_{i,j}/\sqrt{\sigma_{i}^{2}\sigma_{j}^{2}}$
Variance	$\sigma^{2}=[\sum_{i=1}^{N}{\left(x_{i}-\mu \right)}^{2}]/N$	Frequency Center	*FC* = $\sqrt{f_{1}f_{2}}$
Absolute Mean Value	$A_{M}=\mu \left(abs_{X}\right)$	Energy	*E* = $\sum_{i=1}^{N}{\left|x_{i}\right|}^{2}$
SRA	SRA = $\mu \left(\sqrt[{2}]{abs_{X}}\right)$	Entropy	*H* $\left(X\right)=-\sum_{i=1}^{N}p\left(x_{i}\right)\log_{2}p\left(x_{i}\right)\;$
Standard Deviation	$STD=\sqrt{\frac{1}{N-1}\sum_{i=1}^{N}{\left(x_{i}-\bar{x}\right)}^{2}}$	RMS Frequency	${RMS}_{fr}=\mu \left(\sqrt{\left(fr_{x}{}_{1}^{2}+fr_{x}{}_{2}^{2}\ldots +fr_{x}{}_{n}^{2}\right)}\right)$
Zero Crossing Rate	*ZC* = $\sum_{i=1}^{N}\left|sgn\left(x_{i}\right)-sgn\left(x_{i-1}\right)\right|/2N$	Minimum	Min$\left(x_{i}\right)$ i∈{1,…,N}
Median	$M=\left(\frac{n/2-cf}{f}\right)\left(w\right)+L_{m}\;$	Maximum	Max$\left(x_{i}\right)$ i∈{1,…,N}
Interquartile Range	*IQR* = $\frac{3}{4\left(n+1\right)}th\;term-\frac{1}{4\left(n+1\right)}th\;term$	Peak to Peak	*PPV* = Max$\left(x_{i}\right)-\mathrm{Min}\left(x_{i}\right)$
Root Mean Square	$X_{rms}=\sqrt{\frac{1}{N}\left(x_{1}^{2}+x_{2}^{2}+\ldots +x_{n}^{2}\right)}$	Impulse Factor	*IF* = $\frac{x_{peak}}{x_{mean}}$
Correlation coefficient	γ = $\;\;\frac{n\left(\sum xy\right)-\left(\sum x\right)\left(\sum y\right)}{\left[n\sum x^{2}-{\left(\sum x\right)}^{2}\right]\left[n\sum y^{2}-{\left(\sum y\right)}^{2}\right]}$	Margin Factor	*MF* = $x_{peak}/x_{sra}$
Skewness	*SV* = $\frac{1}{N}\sum_{i=1}^{N}{\left(\frac{x_{i}-\bar{x}}{\sigma }\right)}^{3}$	Shape Factor	*SF* = $X_{rms}/\mu \left(abs_{X}\right)$
Kurtosis	*KV* = $\frac{1}{N}\sum_{i=1}^{N}{\left(\frac{x_{i}-\bar{x}}{\sigma }\right)}^{4}$	Crest Factor	*CF* = $\;\frac{\left|x_{peak}\right|}{x_{rms}}$

**Table 2:** Error rates on test data for each activity for the three feature subsets, *T*3, *R*3, and *W*3.

	Walking	Walking Upstairs	Walking Downstairs	Sitting	Standing	Laying
*T*3	18.55%	25.69%	24.05%	19.14%	20.3%	23.28%
*R*3	52.82%	46.71%	32.62%	54.79%	67.86%	42.27%
*W*3	88.51%	100%	100%	99.79%	49.06%	74.86%

**Table 3:** Performance of different feature subsets extracted by BAROQUE from the smartphone and smartwatch sensor data in the WISDM dataset, respectively.

	Smartphone Sensor Data	Smartwatch Sensor Data
Used Activities	clapping, writing, eating soup, climbing stairs, folding clothes, playing catch, dribbling a basketball and kicking a soccer ball	
Selected Features	Feature number of *sfp*: 0,2,3,5,6,7,9,12,14,15,20,21,22,23,25,33, 35,36,37,39,40,42,43,44,45,46,47,49,50,53, 55,56,59,60,61,63,65,66,68,69,70,71,73, 78,80,81,88,89,90,91,92,104,105,109,118, 119,122,125,127,128,129,133,135,136,138, 139,141,145,148,150,152,155,156,159, 161,164,169,171,175,178,179,180,181	Feature number of *sfw*: 0,5,9,10,11,15,16,20,21,27,31,36,45,50,51, 54,58,59,61,64,70,76,80,81,82,85,86,90, 91,96,98,99,105,108,110,111,116,123,125, 126,128,130,131,137,141,145,146,148,150, 151,156,158,160,161,164,166,167,168, 172,178,181
Accuracy using *sfp*	92.22%	93.49%
Accuracy using *sfw*	86.01%	95.35%
Joint Features	0,5,9,15,20,21,36,45,50,59,61,70,80,81,90,91,105,125,128,141,145,148,150,156,161,164,178,181	
Overlap Ratio	0.337 (28/83)	0.459 (28/61)

**Table 4:** Performance comparison among different feature selection algorithms.

Algorithms	Need All Features	Time Cost (s)	Prediction Accuracy (%)
PCA	Yes	1.38	99.65%
*k*PCA	Yes	100.2	99.65%
Relief-F	No	161.74	99.83%
CFS	No	6147.22	95.54%
SFFS	No	160,817.5	99.98%
BAROQUE	No	32.46	99.91%

**Table 5:** Comparison between BAROQUE and other swarm-based algorithms in terms of accuracy.

Dataset	No-FS	GA	BPSO	ACO	BAROQUE
UCI-HAR	97.86%	97.37%	97.48%	97.38%	97.48%
WISDM_W	81.09%	78.60%	80.00%	76.12%	80.93%
WISDM_A	89.30%	86.35%	88.84%	87.91%	88.99%
UT_complex	99.83%	99.83%	99.91%	99.83%	99.91%

**Table 6:** Classification accuracies of different HAR solutions using the UCI-HAR dataset.

	BAROQUE	SMC-SVM	MC-SVM	Convnet	CAT
walking	98.84%	98.99%	99%	98.99%	89.24%
walking downstairs	98.34%	98.33%	98%	100%	100%
walking upstairs	99.37%	97.24%	96%	100%	94.52%
standing	95.98%	97.18%	97%	93.23%	99.19%
sitting	97.94%	97.76%	88%	88.80%	99.08%
laying	100%	99.26%	100%	87.71%	99.12%
Average	98.41%	98.13%	96%	94.79%	96.86%

## Data Availability

UCI-HAR dataset is available at https://archive.ics.uci.edu/ml/datasets/Human+Activity+Recognition+Using+Smartphones (accessed on 31 January 2021). WISDM dataset is available at https://archive.ics.uci.edu/ml/datasets/WISDM+Smartphone+and+Smartwatch+Activity+and+Biometrics+Dataset+Data+Set (accessed on 31 January 2021). And UT_complex dataset is available at https://www.utwente.nl/en/eemcs/ps/research/dataset/ (accessed on 27 March 2021).
